# Supplementary material for: Long Term Neurodevelopmental Outcomes after Sevoflurane Neonatal Exposure of Extremely Preterm Children: A Cross-Sectional Observationnal Study
Source: Children (Basel). 2022 Apr 12;9(4):548. doi: 10.3390/children9040548 (PMC9028040; doi:10.3390/children9040548)

Table S1. Pre-selected explanatory variable used to build the propensity score.

Nosocomial infection	Qualitative variable	Details	1.yes / 2.probable / 3.certain
Anemia	Qualitative variable	Details	0.no / 1.yes
Surgery requirement	Qualitative variable	Details	0.no / 1.yes
Chronic lung diseaese	Qualitative variable	Details	0.no / 1.yes
Patent ductus arteriosus	Qualitative variable	Details	0.no / 1.yes
Retinopathy of prematurity	Qualitative variable	Details	0.no / 1.yes
Antenatal steroid	Qualitative variable	Details	0.no / 1.yes
Gestational age	Quantitative variable	Details	Continuous
Sex	Qualitative variable	Details	1.male / 2.female

Figure S1: Propensity score before and after matching

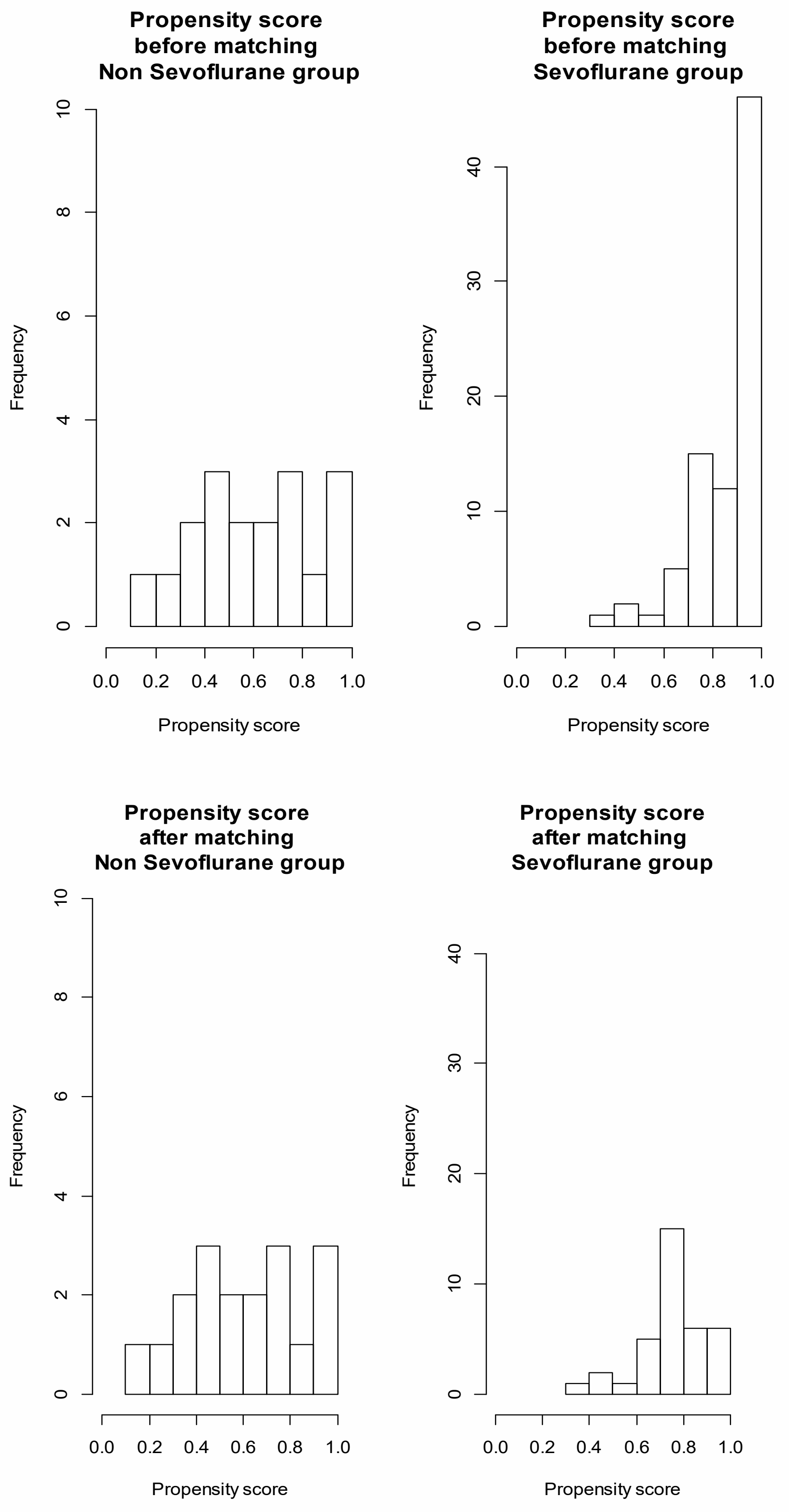

Supplement: Supplementary file 1 [file children-09-00548-s001.zip › children-1619376-supplementary.pdf]
